# Comparison of NOSES and Conventional Laparoscopic Surgery in Colorectal Cancer: Bacteriological and Oncological Concerns

**DOI:** 10.3389/fonc.2020.00946

**Published:** 2020-06-25

**Authors:** Qianhui Ouyang, Jian Peng, Shuai Xu, Jie Chen, Wen Wang

**Affiliations:** ^1^General Surgery Department, Xiangya Hospital, Central South University, Changsha, China; ^2^National Clinical Research Center for Geriatric Disorders, Xiangya Hospital, Central South University, Changsha, China

**Keywords:** natural orifice specimen extraction surgery, conventional laparoscopy, oncological, bacteriological, colorectal cancer, asepsis and tumor-free technique

## Abstract

**Background:** Colorectal natural orifice specimen extraction surgery (NOSES) is considered to be a scarless operation that avoids the laparotomy of extraction specimen, but bacteriological and oncological concerns are raised with this technique.

**Objective:** The purpose of this study was to compare the oncological and bacteriological outcomes of NOSES and conventional laparoscopic (CL) procedures.

**Methods:** This is a retrospective study of prospectively collected outcomes data. Patients operated with colorectal cancer from January 2016 to December 2019 in Xiangya Hospital were assigned to the group NOSES and the group CL according to the size of the tumor. Prior to dissection, peritoneal lavage fluid was collected for cytological assessment. At the end of the procedure, peritoneal lavage fluid was collected for aerobic culture and cytological assessment. Baseline characteristics and short-term and long-term outcomes for NOSES and CL were compared.

**Results:** Between January 2016 and December 2019, 212 patients were enrolled from our center and 185 patients were analyzed (96 and 89 in NOSES and CL groups, respectively). The bacterial positive rate of peritoneal lavage fluid was 34.4 vs. 32.6% in NOSES and CL groups, respectively (*P* = 0.80). The positive rate of tumor cells in peritoneal lavage fluid was 7.3 vs. 9.0% in NOSES and CL groups, respectively (*P* = 0.67). Univariate analysis showed that the positive rate of tumor cells in peritoneal lavage fluid was significantly associated with tumor invasion depth and lymph node metastasis (*P* < 0.05). T4 (OR = 20.47, 95%CI = 1.241–337.661; *P* = 0.04), N1 (OR = 5.445, 95%CI = 1.412–20.991; *P* = 0.01), and N2 (OR = 6.315, 95%CI = 1.458–27.348; *P* = 0.01) served as independent predictors of peritoneal lavage fluid positive oncology patients. Local recurrence-free survival was not significantly different between two groups (HR = 0.909, 95%CI = 0.291–2.840; *P* = 0.87).

**Conclusions:** Compared with conventional laparoscopic procedure, NOSES is in conformity with the principle of asepsis and tumor-free technique and can be worthy of clinical application and promotion.

## Introduction

In recent years, NOSES has drawn wide attention in the treatment of colorectal cancer, which has been considered as an alternative approach to conventional laparoscopic surgery and open surgery for selected patients (1–3). NOSES is another stepping stone toward “incisionless” surgery to reduce pain and wound-related complications. Many studies demonstrated that there were lower analgesic requirements and less pain in NOSES compared with conventional laparoscopic colectomy (4–6). Although the recognition of NOSES in the colorectal field is increasing, there are still concerns about its compliance with the principles of bacteriology and oncology.

One potential risk of NOSES is peritoneal contamination secondary to the opening of the colon or rectal stump for extracting the specimen. During the NOSES procedures, enterotomy, and bowel reconstruction are performed in the abdominal cavity, and anvils are inserted into the abdominal cavity through natural orifice, which may cause bacteriological problems (7, 8). In addition to bacteriological issues, another major issue is the oncological safety of NOSES in colorectal cancer. Extraction of specimens through natural orifice may squeeze tumors, causing tumor cells to fall out of the pelvic or abdominal cavity, which is questionable in terms of oncological safety (2, 9).

The issues of bacteriology and oncology are not only NOSES needs to face, conventional laparoscopic surgery also with these problems. Therefore, we collected postoperative peritoneal lavage fluid for oncological and bacteriological examination, and compared the long-term oncological outcomes of NOSES and CL surgery.

## Materials and Methods

### Patients

A total of 212 patients with colorectal cancer were enrolled in the study from January 2016 to December 2019 at our hospital. After strict inclusion and exclusion criteria, a total of 200 patients met the requirements. The orifice selection for specimen extraction is mainly based on the size of the tumor, especially the maximum circumferential diameter (CDmax). Eligible patients were matched into two study groups based on tumor size and signed informed consent: (1) NOSES (CDmax < 3cm); (2) Conventional laparoscopic surgery (CDmax: 3–5cm). All patients were followed up for postoperative abdominal infection and local tumor recurrence. Patients were followed up regularly after discharge, including tumor marker blood tests and enhanced chest/abdominal/pelvic CT. This study was approved by the Ethics Committee of Xiangya Hospital of Central South University, China (No: 201601021), and all patients provided written informed consent.

The inclusion criteria were as follows: (1) patients aged between 18 and 80 years; (2) histopathology confirmed as colorectal adenocarcinoma; (3) preoperative imaging (CT and MR) assessments showed that colorectal cancer did not penetrate the serosa (≤T3); (4) tumor circumference <5 cm; (5) enhanced chest and abdominal pelvic CT scans before operation excluded liver metastasis, lung metastasis, and other distant organ metastases.

The preoperative exclusion criteria were as follows: (1) tumors could be resected by endoscopic submucosal dissection (ESD) and endoscopic mucosal resection (EMR); (2) body mass index (BMI) > 30 kg/m^2^; (3) patients with severe perforation, bleeding, or obstruction requiring emergency surgery; (4) recurrent cases; (5) patients undergoing neoadjuvant therapy or preoperative radiotherapy; (6) Anesthesiologists (ASA) score ≥ IV; (7) active period of infection; (8) blood neutrophils <3 × 10^9^/L.

The postoperative exclusion criteria were as follows: (1) combined with other parts of surgical or converted to open surgery; (2) preoperative peritoneal lavage fluid tumor cytology test positive; (3) peritoneal metastasis.

### Quality Control of Surgery

To control the quality of the operation, all selected patients' operations were performed by the same group of surgeons in accordance with uniform operating standards. The surgeon and assistants were fixed and had rich experience more than 12 months of practice in NOSES operation. Therefore, it was possible to effectively control the bias caused by different surgical proficiency.

### Surgical Procedure

#### Preoperative Preparation

We took the following bowel preparation for the patients: diet adjustment, semiliquid diet 3 days before surgery, liquid diet 2 days before surgery. From 6 p.m. to 8 p.m. the day before surgery, the bowel preparation was performed with 90 ml of sodium phosphate oral solution mixed well with 1.5 L of water and the patient need to drink the solution in 30 min, and rectal enema with 500 ml normal saline in 10 p.m.

#### Technique

**Figure 1:**
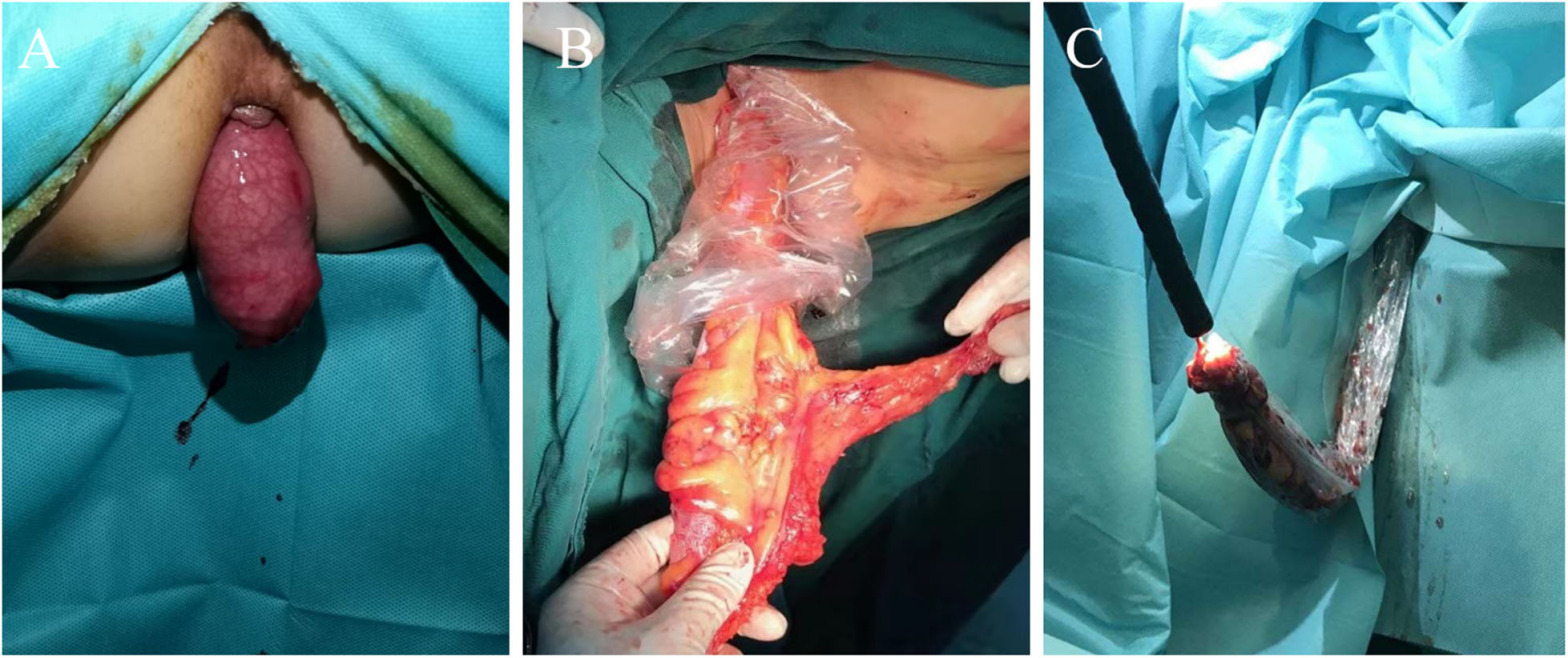
Three methods for extraction specimens. **(A)** Transanal specimen eversion and extra-abdominal resection technique. **(B)** Transluminal specimen extraction and extra-abdominal resection technique. **(C)** Intra-abdominal specimen resection and transluminal extraction technique.

After successful anesthesia, patients were placed in the modified lithotomy position, and an antibiotic prophylaxis (2 g of ceftazidime) was administered prior to incising abdominal skin. Before exploration of the abdominal cavity and mobilization of the tumor, 100 ml of saline solution was instilled in the area adjacent to the tumor, followed by immediate aspiration this lavage fluid for cytological assessment. Abdominal procedures are the same for both groups, according to the CME and TME principles. The difference between the two groups of surgery is the way of specimen extraction. The specimen of group CL was taken through the assisted abdominal wall incision. Group NOSES specimen was extracted through rectal anus. There were different methods for NOSES depending on the location of the colorectal tumor. According to the methods of specimen extraction, NOSES was divided into three categories (2) (Figure 1): (1) Transanal rectal eversion and extra-abdominal resection, this technique was mainly used to lower rectal resection with small tumor. Transected distal stump was retracted by grasping the staple line using a cured clamp and everted out. Rectum was transected distally from the mass by using electrocautery. The proximal closed colonic end in the abdomen was pulled out transanally using a cured clamp under guidance of the laparoscope. The closed end of the colon was opened. Anvil of end-to-end anastomotic stapler was placed in and fixed with purse-string suture. Proximal colon with anvil was sent back into the abdomen. Rectum was closed using a stapler device. Anastomosis was done intracorporeally under laparoscopic guidance using a transanally placed end-to-end circular stapler. Eversion makes it possible to perform resection and placement of the anvil extracorporeally. (2) Transluminal specimen extraction and extra-abdominal resection, this technique was mainly used for middle rectal resection. The rectal wall is cut off at the distal resection line, and the distal side of specimen is gently pulled outside of the patient body transanally. The proximal rectal resection is performed extra-abdominally. The anvil is introduced into the bowel lumen and closed with purse-string suture, and the sigmoid colon is delivered back to pelvic cavity. The open rectal stump is closed by using linear stapler. The circular stapling device is introduced into the rectum, and an end-to-end anastomosis is performed. (3) Intra-abdominal specimen resection and transluminal extraction, this technique was mainly used for upper rectal resection and colectomy. The distal and proximal bowel division is performed using linear stapler. The specimen is extracted through the anus. The open proximal stump is closed with a linear stapler. In colon cancer, the linear stapling device is introduced into the bowel, and functional side to side anastomosis is performed. When the tumor is located in the colon, we need colonoscopy to assist specimen extraction. In rectal cancer, the circular stapling device is introduced into the rectum, and an end-to-end anastomosis is performed. We used a sterile specimen bag to assist transluminal specimen extraction. The aim was to avoid direct contact between the specimen and the natural opening to ensure sterile and tumor-free operation. After bowel anastomosis, 200 ml of saline solution was instilled around the anastomosis, followed by immediate aspiration this lavage fluid with leaving 50 ml for cytological examination and 5 ml for bacteriological examination. Postoperative intravenous infusion of ceftazidime for 3 days.

### Method of Cytological and Bacterial Detecting

We poured the peritoneal lavage fluid sample into a centrifuge tube to centrifuge at 2,500 rpm/min for 10 min and observed the sediment. When there is a lot of sediment, we discarded the supernatant and used a pipette to aspirate the cell layer to make a conventional smear. After drying and fixing with 95% alcohol for 15 min, stain with hamatoxylin and eosin (H &E) was made; When the precipitate is small, we discarded the supernatant, added 30 mL of Cytolyt solution to the remaining sample, shaked on a shaker for 10 min, centrifuged at 1,500 rpm/min for 10 min, discarded the supernatant, and transfered the precipitate to Presevcyt preservation solution vial. After standing for 20 min, we made a liquid-based smear in a Thinprep 5000 instrument, fixed with 95% alcohol for 15 min and stained with HE, and then viewed with an optical microscope to find exfoliated cancer cells. The sample was considered positive if at least one tumor cell was detected. Otherwise, it is negative. The peritoneal lavage smears were checked by two pathologists who would have to agree on the results.

Specimens were inoculated on blood agar plates for incubation, culture, and purification. The drug sensitivity test was performed using the K–B disk method, and the bacteria were identified using the Micro Scan Walk Away 40S system.

### Follow-Up

All patients were followed up in an outpatient clinic. Adjuvant chemotherapy with oxaliplatin and capecitabine for 6 months was recommended for patients who had complete tumor resection and pathological stage II with risk factors and all stage III tumors. Patients were assessed by physical examination and analysis of tumor markers every 3 months for the first 2 years and then every 6 months thereafter until 5 years after surgery. Chest and abdominal pelvic CT was performed every 6 months for the first 3 years after surgery and once a year thereafter.

### Statistical Analyses

The results were analyzed with the SPSS (version 25) program. The quantitative data were expressed as the mean ± SD and were compared using an independent samples *t*-test. Qualitative data were compared using the χ^2^ test or Fisher's exact test. To determine the risk factors for positive of oncology and positive of bacteriology, a univariate analysis was first performed using χ^2^ test or Fisher's exact tests. Subsequently, a multivariate analysis was conducted using a logistic regression model that included all variables at *P* < 0.05 in the univariate analysis. The survival curves were plotted by Kaplan–Meier method. Whether there was a statistically significant difference in the local recurrence-free survival between two groups was detected via Log-rank test. *P* < 0.05 suggested that the difference was statistically significant.

## Results

**Figure 2:**
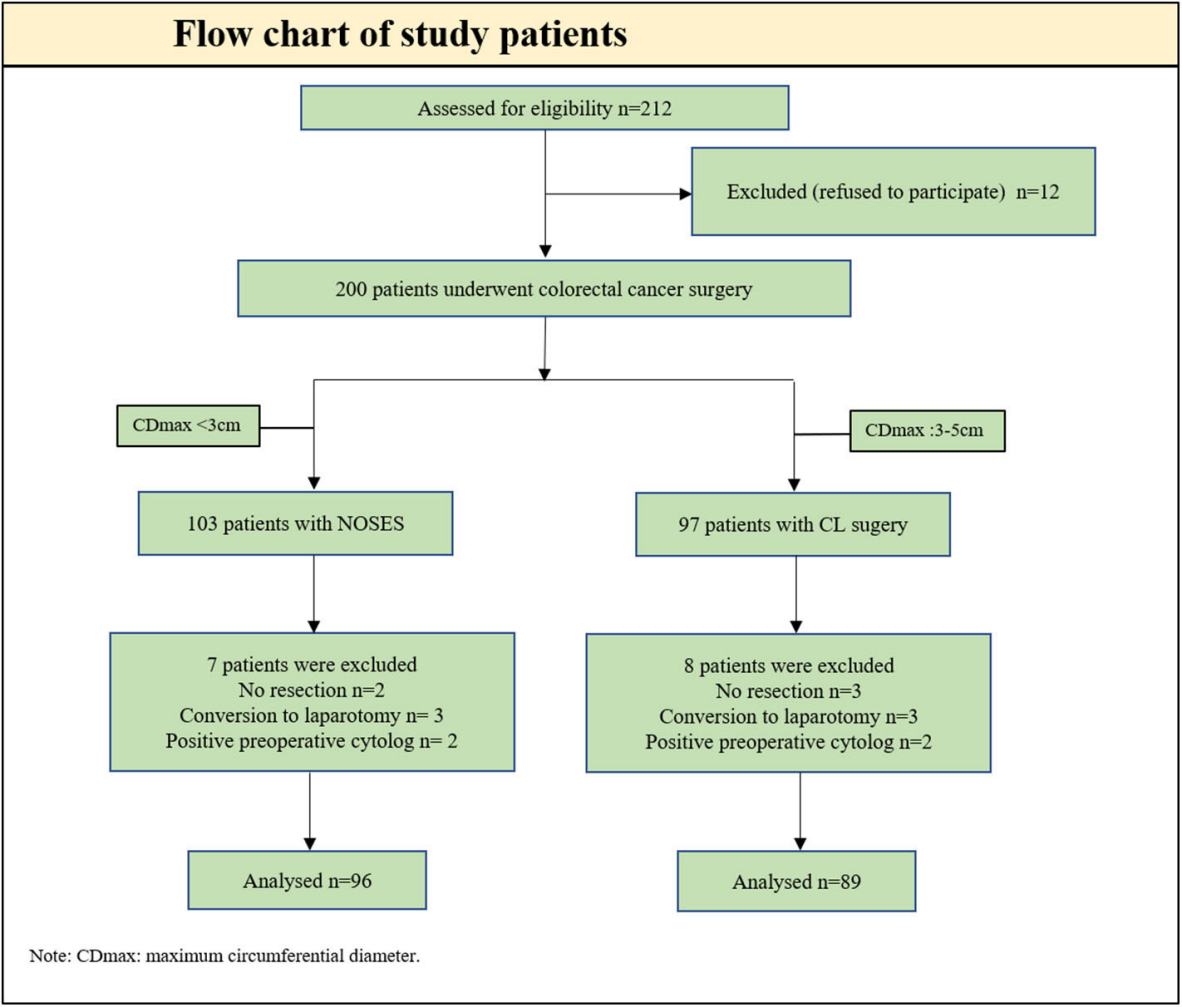
Flow chart of study patients. CD max, maximum circumferential diameter.

From January 2016 to December 2019, 200 patients underwent colorectal cancer surgery, including NOSES (103 patients) and group CL (97 patients). Excluded from the analysis were five patients with peritoneal metastasis (no resection: two in group NOSES and three in group CL), six patients with conversion to open laparotomy (three in group NOSES and three in group CL), and four patients with positive preoperative cytology (two in group NOSES and two in group CL). As a result, 185 patients were included in the analysis, 96 in group NOSES, and 89 in group CL (Figure 2). Patients undergoing NOSES all extract specimen through the anus.

### Patient Characteristics

In the present study, 185 patients with colorectal cancer were enrolled, including 93 males (50.3%) and 92 females (49.7%), aged 23–80 years (mean 59.29 ± 11.35). The mean BMI was 22.71 ± 2.79 kg/m^2^. Comparison of baseline characteristics between the patients who underwent NOSES and conventional laparoscopic surgery is shown in Table 1. There were no statistically significant differences in the age, gender, BMI, ASA score, CEA, cT category (rectum), tumor location, invasion depth, nodal metastasis, TNM stage, venous invasion, and neurological invasion between the two groups (*p* > 0.05).

**Table 1:** Comparison of baseline characteristics in patients with colorectal cancer between the group NOSES and Conventional Laparoscopic Surgery (CL) group.

**Characteristics**	**NOSES (*N* = 96)**	**CL (*N* = 89)**	***P***
Age (M ± SD), years	58 ± 11	61 ± 11	0.11
BMI (kg/m^2^)	22.5 ± 2.9	22.9 ± 2.6	0.38
**Gender**			0.21
Male	44 (45.8%)	49 (55.1%)	
Female	52 (54.2%)	40 (44.9%)	
**ASA score**			0.99
1	13 (13.5%)	12 (13.5%)	
2	25 (26.0%)	24 (26.9%)	
3	58 (60.5%)	53 (59.6%)	
**CEA (ng/mL)**			0.1
<5	75 (78.1%)	60 (67.4%)	
≥5	21 (21.9%)	29 (32.6%)	
**cT category (rectum)**			0.79
cT1	11 (13.6%)	7 (11.3%)	
cT2	34 (42.0%)	24 (38.7%)	
cT3	36 (44.4%)	31 (50.0%)	
**Tumor location**			0.37
Ascending colon	1 (1.0%)	2 (2.2%)	
Transverse colon	1 (1.0%)	3 (3.4%)	
Descending colon	3 (3.1%)	3 (3.4%)	
Sigmoid colon	10 (10.4 %)	19 (21.3%)	
Rectosigmoid colon	24 (25.0%)	17 (19.1%)	
Mid rectum	42 (43.8%)	33 (37.1%)	
Lower rectum	15 (15.6%)	12 (13.5%)	
**Invasion depth (T factor)**			0.59
T0–T1	8 (8.3%)	4 (4.5%)	
T2	14 (14.6%)	18 (20.2%)	
T3	71 (74.0%)	64 (71.9%)	
T4	3 (3.1%)	3 (3.4%)	
**Nodal metastasis (N factor)**			0.68
N0	64 (66.7%)	54 (60.7%)	
N1	21 (21.9%)	22 (24.7%)	
N2	11 (11.5%)	13 (14.6%)	
**TNM stage**			0.49
0–I	16 (16.7%)	10 (11.2%)	
II	48 (50.0%)	44 (49.4%)	
III	32 (33.3%)	35 (39.3%)	
**Venous invasion**			0.21
Yes	11 (11.5%)	16 (18.0%)	
No	85 (88.5%)	73 (82.0%)	
**Neurological invasion**			0.18
Yes	8 (8.3%)	13 (14.6%)	
No	88 (91.7%)	76 (85.4%)	

*CT category, clinical tumor invasion depth*.

### Comparison of Postoperative Indexes

Postoperative outcomes in Table 2. Peritoneal cytology with the Thin-prep method was positive in seven cases (7.3%) in group NOSES, while there were eight cases (9.0%) in group CL. There were no statistically significant differences in the oncological outcomes between the two groups (*p* > 0.05). In terms of bacteriological outcomes, 33 cases (34.4%) were positive in group NOSES, while 29 cases (32.4%) were positive in group CL. There were no statistically significant differences in the bacteriology outcomes between the two groups (*p* > 0.05). Detection of WBC, PTC, CRP, and other infection-related indicators on the first day after operation, no significant difference between the two groups. Moreover, the intraperitoneal infection and postoperative temperature had no statistically significant differences between the two groups (*p* > 0.05). Compared with the CL group, patients in the NOSES group had a lower rate of additional analgesic use (9.4 vs. 32.6%, *p* < 0.001), lower postoperative pain score (*P* < 0.001), shorter hospital stay (7.03 ± 1.30 vs. 9.37 ± 2.52, *p* < 0.001) and incision-related complications rate was lower (0 vs.5.6%, *p* = 0.02).

**Table 2:** Comparison of postoperative outcomes in patients with colorectal cancer between the group NOSES and Conventional Laparoscopic Surgery (CL) group.

**Variables**	**NOSES (96)**	**CL (89)**	***P***
**Postoperative oncological outcomes**			0.67
Negative	89 (92.7%)	81 (91.0%)	
Positive	7 (7.3%)	8 (9.0%)	
**Bacteriological Outcomes**			0.8
Negative	63 (65.6%)	60 (67.4%)	
Positive	33 (34.4%)	29 (32.6%)	
**Postoperative WBC (1d)**			0.53
<10 × 10^9^ g/L	53 (55.2%)	45 (50.6%)	
≥10 × 10^9^ g/L	43 (44.8%)	44 (49.4%)	
**PTC (1d)**			0.35
<0.25 ng/ml	77 (80.2%)	68 (76.4%)	
≥0.25 ng/ml	19 (19.8%)	21 (23.6%)	
**CRP (1d)**			0.91
<8 mg/L	74 (77.1%)	68 (76.4%)	
≥8 mg/L	22 (22.9%)	21 (23.6%)	
**Temperature**			0.21
<38.5°C	85 (88.5%)	73 (82.0%)	
≥38.5°C	11 (11.5%)	16 (18.0%)	
**VAS score**			
Day 1 postoperatively	2.43 ± 0.87	5.34 ± 1.02	<0.001
Day 3 postoperatively	1.43 ± 0.81	3.67 ± 0.84	<0.001
Day 5 postoperatively	0.96 ± 0.75	2.35 ± 0.91	<0.001
Intraperitoneal infection	4 (4.2%)	3 (3.4%)	1
Usage rate of additional analgesics	9 (9.4%)	29 (32.6%)	<0.001
Incision-related complications	0	5 (5.6%)	0.02
Postoperative hospital stay (d)	7.03 ± 1.30	9.37 ± 2.52	<0.001

### Analysis of Risk Factors for Oncology Positive

We performed univariate and multivariate analyses of the clinical and pathological variables which could potentially influence the results of oncology (Table 3). In the univariate analysis, the invasion depth and nodal metastasis were significantly associated with positive peritoneal lavage fluid oncology. The incidence of oncology positive in T4 with invasion depth was significantly higher than in T3 and T0–T2 (33.3 vs. 8.9 vs. 2.3%, *P* < 0.05). The incidence of oncology positive in N2 and N1 with nodal metastasis was significantly higher than in N0 (20.8 vs. 14.0 vs. 3.4%, *P* < 0.05). In the multivariate analysis, the nodal metastasis (N1, N2) and invasion depth (T4) were an independent risk factor for positive peritoneal lavage fluid oncology.

**Table 3:** Univariate and multivariate analysis of clinical and pathological factors for oncology of peritoneal lavage fluid.

**Factors**	**Number**	**Univariate analysis**		**Multivariate analysis**	
		**Oncology positive rate (%)**	***P***	**Odds ratio**	***P***
**Gender**			0.77		
Male	93	7.5			
Female	92	8.7			
**Age (years)**			0.63		
<60	85	7.1			
≥60	100	9.0			
**CEA (ng/mL)**			0.55		
<5	135	7.4			
≥5	50	10.0			
**cT category (rectum)**			0.25		
cT1	18	9.0			
cT2	58	5.2			
cT3	67	16.7			
**Tumor location**			0.79		
Colon	42	7.1			
Rectum	143	8.4			
**Invasion depth (T factor)**			0.04		
T0–T2	44	2.3		1	
T3	135	8.9		4.699 (0.580–38.067)	0.15
T4	6	33.3		20.470 (1.241–337.661)	0.04
**Nodal metastasis (N factor)**			0.003		
N0	118	3.4		1	
N1	43	14.0		5.445 (1.412–20.991)	0.01
N2	24	20.8		6.315 (1.458–27.348)	0.01
**Venous invasion**			0.89		
Yes	27	7.4			
No	158	8.2			
**Neurological invasion**			0.39		
Yes	21	14.3			
No	164	7.3			

### Follow-Up Results of Patient's Local Recurrence-Free Survival

**Figure 3:**
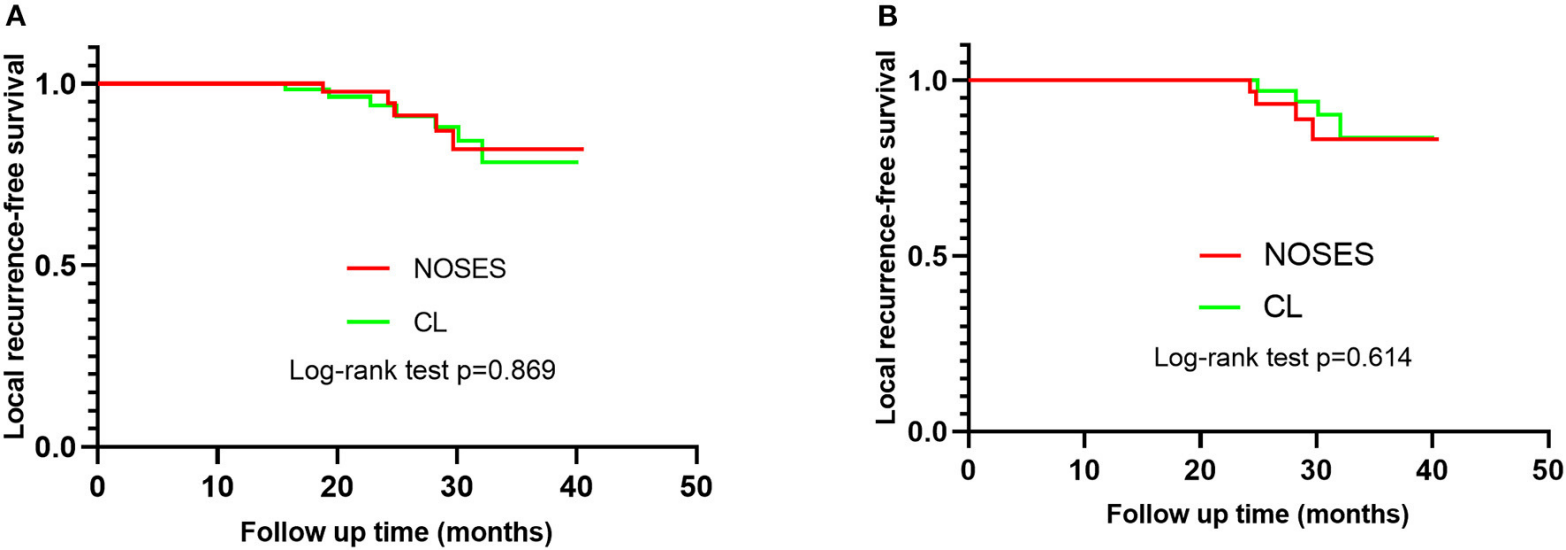
Kaplan–Meier survival curve of patients in NOSE and group CL. **(A)** The difference of the local recurrence-free survival rate of patients in the two group has no statistical significance (*p* = 0.87), Hazard ratio 0.909, (95%CI = 0.291–2.840). **(B)** Sixty-six patients were followed for more than 2 years. The difference of the local recurrence-free survival rate of patients in the two group has no statistical significance (*p* = 0.61).

The patients were followed up for 4–41 months. In group NOSES, the tumor relapsed in five patients at 30, 28, 25, 24, and 19 months after operation, and the recurrence rate was 5.2% (5/96). In group CL, the tumor relapsed in seven patients at 16, 19, 23, 24, 28, 30, and 32 months after operation, and the recurrence rate was 7.9% (7/89). The recurrence rate of tumor had no significant difference between the two groups (*p* = 0.56). The Kaplan–Meier local recurrence-free survival of group NOSES and group CL are shown in Figure 3, and the log-rank test revealed that the local recurrence-free survival rate had no statistically significant difference between the two groups (*p* = 0.79). Sixty-six patients were followed for more than 2 years, including NOSES (32 patients) and group CL (34 patients). There was no significant difference in local recurrence rate between the two groups (*p* = 0.93).

### Analysis of Bacterial Species

The contamination rate of peritoneal lavage fluid was 34.4 vs. 32.6% in group NOSES and group CL, respectively. Gram-negative bacteria were the main bacteria in two groups of peritoneal lavage fluid including *Escherichia coli, Enterobacter cloacae, Pseudomonas aeruginosa, Acinetobacter reesei, Klebsiella pneumoniae, Enterobacter aerogenes, Citrobacter freundii, Aeromonas caviae*, and *Aeromonas* Vickers. Among them, *E. coli* is the main detected bacteria in line with bowel flora. Table 4 showed details of the types of bacteria cultured by the two groups of peritoneal lavage fluid.

**Table 4:** Bacterial types in group NOSES and group CL with bacteriology positive.

**Organism**	**NOSES (33)**	**CL (29)**	**No. (62)**
*Escherichia coli*	22	21	43
*Enterobacter cloacae*	1	1	2
*Pseudomonas aeruginosa*	1	0	1
*Enterococcus faecium*	3	1	4
*Corynebacterium*	1	0	1
*Streptococcus oralis*	2	1	3
*Acinetobacter reesei*	1	0	1
*Klebsiella pneumoniae*	1	1	2
*Enterobacter aerogenes*	1	1	2
*Citrobacter freundii*	0	1	1
*Aeromonas* Vickers	0	1	1
*Aeromonas caviae*	0	1	1

## Discussion

In 1993, Franklin et al. (10) were the first to publish a case of patient who underwent sigmoid resection with transrectal specimen extraction. In recent years, more and more people have noticed that NOSES is more minimally invasive than conventional laparoscopic surgery and has accelerated the postoperative recovery of patients (11–13). It has caused widespread concern in the treatment of colorectal cancer and could be the next step in minimizing minimally invasive surgery (1, 5, 14). However, there is no systematic discussion on whether NOSES operation adds oncological and bacteriological issues. Ngu and Wong (9) reported that five patients with NOSES had no tumor cells found in the peritoneal lavage fluid. Costantino et al. (15) showed the contamination rate of peritoneal fluid was 100 vs. 88.9% in NOSE and non-NOSE procedures. The high contamination rate likely because they did not use a sterile specimen bag to protect the resected specimen during the operation. Our research strengthened the control of aseptic and tumor-free operation and increased the sample size. The use of sterile protective sleeves to reduce tumor cell planting and bacterial contamination of the abdominal cavity.

In this study, our data showed that there were no statistically significant difference in oncology between NOSES and conventional laparoscopic surgery for colorectal cancer. Hence, we think that oncological issue has nothing to do with the surgical approach. We further performed univariate and multivariate analysis on 15 patients with oncology positive. Our study found that the tumor invasion depth and nodal metastasis were independent risk factors for oncology positive. The pT4, pN1, and pN2 increased the risk of postoperative peritoneal lavage fluid oncology positive by 20.47, 5.45, and 6.32 times, respectively. Noura et al. (16) and Temesi et al. (17) showed that the chance of malignant cells being present in the lavage fluid increased as the depth of tumor invasion increased. This showed that the positive rate of cancer cells in the postoperative peritoneal lavage fluid was related to the stage of the tumor itself. Although we used sterile specimen bag during specimen extraction to avoid tumor implantation and peritoneal contamination, there are still tumor cells in the peritoneal lavage fluid of NOSES. Probably because live tumor cells that have the potential to proliferate and possibly metastasize have shed from the primary site before or during surgical resection. Other studies showed the positive rate of postoperative peritoneal lavage fluid in colorectal cancer patients is between 0 and 52% (17–20), the worse the tumor stage, the higher positive rate of tumor cells in peritoneal lavage fluid. The low rate of positive samples in our study may be due to our having only included early stage patients undergoing scheduled curative surgery. This also explains why our patients with NOSES had a lower positive rate of cancer cells in the peritoneal lavage fluid, and did not increase the probability of pelvic and abdominal implantation of cancer cells.

Bacterial contamination of the peritoneal cavity is frequent in colorectal laparoscopic procedures (21), but it is unclear whether NOSES causes increased levels of contamination. We found that the bacterial culture results of the peritoneal lavage fluid collected during NOSES showed a 34.4% positive rate of bacteriology, which was no significantly different from the results of the group CL. There was also no significant difference in the incidence of intraoperative abdominal infection between the two groups after surgery. The higher bacteriological positive rate after surgery is mainly due to the large intestinal flora in the colorectum (22). The bacterial culture results of our peritoneal lavage fluid showed that they were mainly Gram-negative bacteria such as *Escherichia coli*. No epidermal colony was found, which proves that we did not bring in external bacteria when we placed an anvil. The international consensus of NOSES suggests that prophylactic antibiotics should be used before surgery, perfect bowel preparation, intraperitoneal irrigation during operation, anal lavage with a large amount of povidone iodine and normal saline, use of transluminal wound protector and placement of pelvic or abdominal drainage tube to reduce the bacterial load of NOSES (1, 23). We perform NOSES procedures in accordance with specifications, which will not increase the incidence of bacterial contamination and abdominal infections.

We compared the tumor cytology and bacterial aerobic culture results of peritoneal lavage fluid in patients with NOSES and patients with conventional laparoscopic surgery, and the results of local recurrence of tumor in two groups were followed up for a long time. The most important finding of our research is that NOSES will not increase tumor implantation and abdominal contamination. Liu et al. (24) analyzed 14 studies through meta-analysis and demonstrated that compared with CL surgery, NOSES may be a safe operation and can achieve similar oncology results. Our conclusions further provide reliable evidence that NOSES meets the expectations of tumor safety. We consider that NOSES is feasible and safe for colorectal cancer surgery, and that it can achieve satisfactory clinical outcomes without noticeable scars in carefully selected patients.

The present study has several limitations. Firstly, the preoperative evaluation of tumor invasion mainly depends on the imaging data. To a certain extent, it depends on the judgment of the imaging doctor and the chief surgeon, which sometimes deviates from the pathological results. Our 185 patients were studied according to the inclusion criteria. The depth of postoperative pathological invasion in six patients was T4, which was different from the selection criteria and increased the positive rate of oncology of the abdominal lavage fluid to a certain extent. Second, the number of patients was not large enough, only 185 patients being ultimately enrolled. A larger population study is need to further confirm our results. Third, some patients have a shorter follow-up time, which may ultimately reduce the relapse rate. Forth, several papers are pointing at size as a prognostic indicator, grouping by tumor size may affect research results, but there is no definite conclusion about whether the size of the tumor diameter affects the prognosis. Finally, oncological and bacteriological problems are caused by many factors, our research can only show that there is no difference between NOSES and conventional laparoscopic surgery in this regard.

In our opinion, we screen patients strictly according to the scope of application of NOSES, employing specimen bags and wound protectors to reduce the possibility of bacterial contamination and tumor cell metastasis (1). NOSES has no significant differences in bacteriology and oncology compared with conventional laparoscopic surgery. Therefore, NOSES is safe and feasible, and can be carried out safely for the right patient. In the next step, we can continue to expand the NOSES sample size and follow up its 5-year overall survival (OS) and disease-free survival (DFS).

## Conclusion

There were no significant differences of bacteriological and oncological results in peritoneal lavage fluid between NOSES and conventional laparoscopic surgery, as well as in long-term oncological outcomes. NOSES did not increase postoperative pelvic and abdominal infections or promote tumor cell planting and metastasis. It is conformed to the principle of asepsis and tumor-free and worthy of clinical application and promotion.

## Data Availability Statement

All datasets generated for this study are included in the article/supplementary material.

## Ethics Statement

The studies involving human participants were reviewed and approved by Ethics Committee of Xiangya Hospital of Central South University. The patients/participants provided their written informed consent to participate in this study.

## Author Contributions

JP and QO designed the study. JP, QO, WW, and SX collected and analyzed data. QO wrote the paper. JP, SX, and JC revised the paper. All authors contributed to the article and approved the submitted version.

## Conflict of Interest

The authors declare that the research was conducted in the absence of any commercial or financial relationships that could be construed as a potential conflict of interest.
